# METHODS TO CONSTRUCT AND ANALYZE COGNITIVE FUNCTION TRAJECTORIES USING CLAIMS AND ASSESSMENT DATA

**DOI:** 10.1093/geroni/igae098.2180

**Published:** 2024-12-31

**Authors:** Olga Jarrín, Haiqun Lin

**Affiliations:** Chair; Co-Chair

## Abstract

This symposium presents the methodology for construction and analysis of the cognitive function change over time during the last five years of life among Medicare beneficiaries. Understanding the changes in cognition is important in maintaining functional independence in last years of life. The first presentation explains the methodology to harmonize data from healthcare claims and clinical assessments across various post-acute and long-term care settings for Medicare beneficiaries who died in 2019, age 50 or older at death, with at least five years of continuous Medicare enrollment. Cognitive function data were harmonized to create a four-category variable: no impairment, memory loss, mild cognitive impairment (MCI), and dementia/severe cognitive impairment. The second presentation examines change in cognitive function over the last five years using the group-based trajectory model for ordered categorical outcomes, revealing ten cognitive function trajectory groups. Sociodemographic and clinical factors were assessed for their association with the trajectories and used to predict trajectory class clusters with machine learning algorithms. The third presentation presents a novel application of the three-step Bolck-Croon-Hagenaars (BCH) method with mixed-effects group-based trajectory model to account for the individual heterogeneous effects, and assess the association between the group membership and risk factors. The fourth presentation leverages the strengths of real-world data for nearly two million Medicare beneficiaries to unpack within-group racial/ethnic and sex differences in cognitive function trajectories and their predictors with discussion from a life-course perspective. Collectively, these methods and findings advance our understanding of differential late-life cognitive decline patterns, informing future etiological and intervention studies.

